# Postglacial species displacement in *Triturus* newts deduced from asymmetrically introgressed mitochondrial DNA and ecological niche models

**DOI:** 10.1186/1471-2148-12-161

**Published:** 2012-08-30

**Authors:** Ben Wielstra, Jan W Arntzen

**Affiliations:** 1 Naturalis Biodiversity Center, P.O. Box 9517, 2300 RA, Leiden, The Netherlands; 2 Faculty of Geo-Information Science and Earth Observation – ITC, University of Twente, P.O. Box 6, 7500 AA, Enschede, The Netherlands

**Keywords:** Contact zone, Ecological niche modeling, Historical biogeography, Phylogeography

## Abstract

**Background:**

If the geographical displacement of one species by another is accompanied by hybridization, mitochondrial DNA can introgress asymmetrically, from the outcompeted species into the invading species, over a large area. We explore this phenomenon using the two parapatric crested newt species, *Triturus macedonicus* and *T. karelinii*, distributed on the Balkan Peninsula in south-eastern Europe, as a model.

**Results:**

We first delimit a ca. 54,000 km^2^ area in which *T. macedonicus* contains *T. karelinii* mitochondrial DNA. This introgression zone bisects the range of *T. karelinii*, cutting off a *T. karelinii* enclave. The high similarity of introgressed mitochondrial DNA haplotypes with those found in *T. karelinii* suggests a recent transfer across the species boundary. We then use ecological niche modeling to explore habitat suitability of the location of the present day introgression zone under current, mid-Holocene and Last Glacial Maximum conditions. This area was inhospitable during the Last Glacial Maximum for both species, but would have been habitable at the mid-Holocene. Since the mid-Holocene, habitat suitability generally increased for *T. macedonicus*, whereas it decreased for *T. karelinii*.

**Conclusion:**

The presence of a *T. karelinii* enclave suggests that *T. karelinii* was the first to colonize the area where the present day introgression zone is positioned after the Last Glacial Maximum. Subsequently, we propose *T. karelinii* was outcompeted by *T. macedonicus*, which captured *T. karelinii* mitochondrial DNA via introgressive hybridization in the process. Ecological niche modeling suggests that this replacement was likely facilitated by a shift in climate since the mid-Holocene. We suggest that the northwestern part of the current introgression zone was probably never inhabited by *T. karelinii* itself, and that *T. karelinii* mitochondrial DNA spread there through *T. macedonicus* exclusively. Considering the spatial distribution of the introgressed mitochondrial DNA and the signal derived from ecological niche modeling, we do not favor the hypothesis that foreign mitochondrial DNA was pulled into the *T. macedonicus* range by natural selection.

## Background

**Figure 1:**
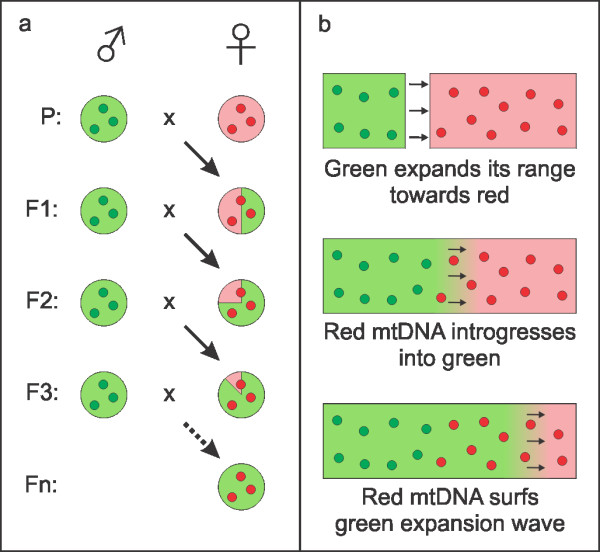
**A schematic representation of asymmetric mitochondrial DNA introgression via hybridization and species displacement.** Figure 1a depicts the transfer of mitochondrial DNA across the species boundary via introgressive hybridization. Large circles reflect the nuclear DNA composition of individuals, small ones their mitochondrial DNA type. There is an initial hybridization event between the members of two species, a red female and a green male. The F1 offspring contain a mix of red and green nuclear DNA, but only red mitochondrial DNA (due to mitochondrial DNA’s matrilineal transmission). Subsequent backcrossing of admixed females with green males over the generations dilutes the red nuclear DNA out, in effect reconstituting the green species’ nuclear genome, but with red mitochondrial DNA (ensured by mitochondrial DNA’s clonal transmission). Figure 1b shows how the outcompeting of one species by another can result in asymmetric mitochondrial DNA introgression. Small circles now reflect the spatial distribution of mitochondrial DNA type and the background the geographical nuclear DNA composition of the population. At the top, the ranges of a red and a green species are in allopatry, but green expands its range towards red. In the middle, green and red have become parapatric. The species hybridize at the contact zone, where red mitochondrial DNA introgresses into the green species (as in Figure 1a). At the bottom, green shifts its distribution further to the right, at the expense of red. As the members of the green species leading the expansion contain red instead of green mitochondrial DNA, only red mitochondrial DNA spreads in the region where the green species displaces the red species. This figure is based on [3,5].

Speciation involves the evolution of reproductive isolating mechanisms, preventing diverging gene pools from merging [1]. If, however, interspecific hybridization still occurs, the stage is set for the transfer of segments of DNA across the species boundary [2-5]. In the majority of eukaryotes, mitochondrial DNA is passed clonally through the female line only and therefore either gets transmitted to the next generation in its entirety or not at all. Mitochondrial DNA does, hence, not ‘dilute’ via recombination over the generations, as is the case with nuclear DNA. [6,7]. Through an initial hybridization event and subsequent backcrossing of female progeny with the paternal species, mitochondrial DNA can spread on the foreign species background (Figure 1a).

Parapatric species can show geographically asymmetric introgression, with one species showing genes typical of the other over a significant part of its range [reviewed in ref. [4]. This could be caused by natural selection, pulling favorable foreign genes into a species’ range [8]. However, empirical and simulation studies show that if displacement of one species by another coincides with hybridization, introgression of neutral genes from the native to the invading species is the expected outcome [4,5]. Furthermore, markers showing reduced intraspecific gene flow (such as mitochondrial DNA) are particularly susceptible to introgression, as their dispersal limitation reduces the chances of being genetically swamped [4,5].

We explain the principle of introgression due to species displacement using mitochondrial DNA as an example (Figure 1b). The rationale is as follows: mitochondrial DNA of a common, native ‘donor’ species is captured by an initially rare invader, through introgressive hybridization. As the invader expands its range at the expense of the native species, its population increases and the introgressed mitochondrial DNA is co-amplified. Consequently, the introgressed mitochondrial DNA surfs the wave of advance and is left in its wake. Initial introgression does not have to occur at a high frequency to result in a mismatch between species identity and mitochondrial DNA type spanning an extensive area.

The climate oscillations related to the glacial/interglacial cycles of the Quaternary ice age repeatedly forced species to shift their distributions [9]. Distribution rearrangement of closely related species after postglacial secondary contact is considered a likely driver of introgression [3,4]. To explore the role of postglacial displacement in introgression, ecological niche modeling - the approximation of the ecological requirements of a species based on the range of environmental conditions experienced at known localities [10] - can be used. By extrapolating ecological niche models on both current and past climate layers, distribution shifts can be identified [11]. By determining which part of the current distribution was uninhabitable at the Last Glacial Maximum (~21 Ka), the area colonized post-glacially can be approximated. Introgression of neutral genes after species displacement would likely be restricted to this particular region, whereas such a geographical limitation would not apply to introgression by positive selection.

### Model system: Triturus newts

**Figure 2:**
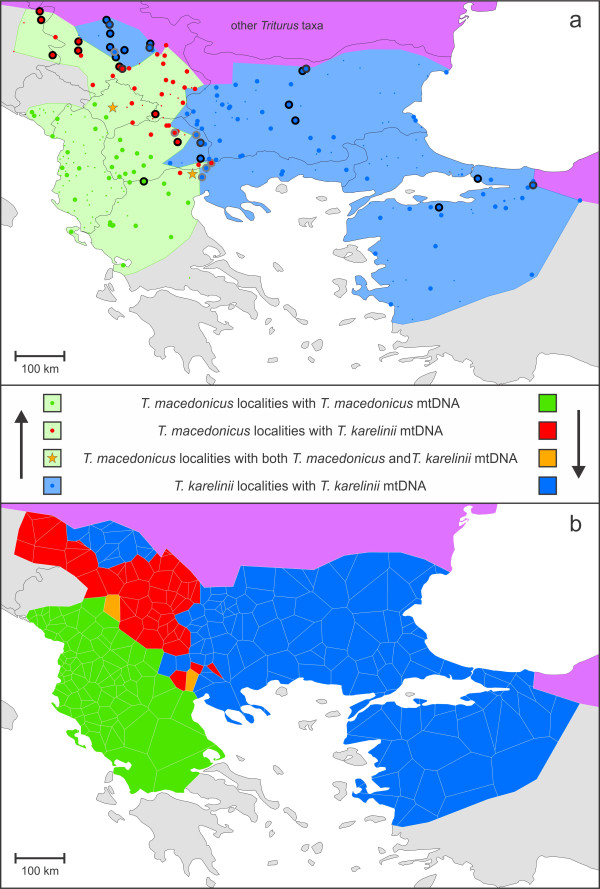
**Distribution and sampling of *Triturus macedonicus* and *T. karelinii* and the zone of asymmetric introgression of *T. karelinii* mitochondrial DNA.** Figure 2a shows the distribution of *T. macedonicus* (shaded green) and *T. karelinii* (blue). Note that the northwestern part of the range of *T. karelinii* is disconnected from the main range (i.e. it constitutes an enclave). Ranges are limited by sea (white), uninhabited land (grey) and other *Triturus* taxa (purple). Green dots represent *T. macedonicus* localities containing original *T. macedonicus* mitochondrial DNA, blue dots *T. karelinii* localities containing *T. karelinii* mitochondrial DNA, red dots *T. macedonicus* localities containing *T. karelinii* mitochondrial DNA and two orange stars *T. macedonicus* localities containing both original and introgressed mitochondrial DNA. Large dots represent localities for which mitochondrial DNA was sequenced and small dots additional localities included in ecological niche modeling. Localities where genetic admixture of the nuclear genome [based on allozyme data presented in Arntzen et al. submitted] is absent are denoted with a thick black border and those where at least one individual is present showing signs of nuclear genetic admixture are denoted with a thick grey border. Localities for which the composition of the nuclear genome could not be consulted were identified based on diagnostic morphological characters. Figure 2b shows the geographical distribution of mitochondrial DNA based on Thiessen polygons, where each polygon covers the area that is closer to its corresponding locality than to another one. Color codes are the same as in Figure 2a. The red and orange polygons were combined to delimit the zone of asymmetric mitochondrial DNA introgression.

We use the two parapatrically distributed crested newt species *Triturus macedonicus* and *T. karelinii* as a model system. The contact zone of these newts is situated on the Balkan Peninsula in southern Europe (Figure 2a). Note that *T. karelinii* comprises three distinct mitochondrial DNA lineages, which might better be regarded as different species [12,13]. When we refer to *T. karelinii*, in this paper, we mean the lineage that occurs in the Balkans and western Turkey, in parapatry with *T. macedonicus*. Their parapatric distribution suggests the two crested newt species, at least locally, are able to competitively exclude one another. This is likely to be driven by different abiotic preferences: *T. macedonicus* is presumed to be relatively more aquatic then *T. karelinii*[13,14].

*Triturus macedonicus* and *T. karelinii* are not sister groups: their most recent common ancestor is the ancestor of all crested newt species (the *T. cristatus* superspecies), estimated to have lived c. 10–11 million years ago [13,15,16]. Analysis of a suite of allozyme markers has shown the nuclear gene pools of *T. macedonicus* and *T. karelinii* to have remained isolated [17,18], Arntzen et al. submitted]. The mitochondrial DNA of the two species is highly distinct [13]. Furthermore, *T. macedonicus* and *T. karelinii* can be distinguished by morphology, including meristic, morphometric and qualitative characters [14,19].

Limited admixture of the nuclear genome does occur at syntopic populations at the contact zone of *T. macedonicus* and *T. karelinii* [Arntzen et al. submitted] (Figure 2a). Furthermore, geographical displacement by *T. macedonicus* has been invoked to explain an apparent enclave of *T. karelinii*, cut off from the main distribution range [19]. These factors would facilitate asymmetric introgression. Indeed, mitochondrial DNA typical of *T. karelinii* has been found in *T. macedonicus*, at localities removed from the current contact zone [20,21]. Here we further explore the introgression of *T. karelinii* mitochondrial DNA into *T. macedonicus*. We first delimit the zone of *T. karelinii* mitochondrial DNA asymmetrically introgressed into *T. macedonicus* through a dense phylogeographic survey. We then build ecological niche models for both species and project these on current, mid-Holocene (~6 Ka) and Last Glacial Maximum (~21 Ka) climate layers. Inferred distribution shifts across the current zone of asymmetric mitochondrial DNA introgression suggest post-glacial displacement of *T. karelinii* by *T. macedonicus*.

## Results

As *T. macedonicus* and *T. karelinii* mitochondrial DNA differs considerably (D_XY_ = 0.091), sequenced individuals could be unambiguously assigned to mitochondrial DNA type (details in Additional file 1). Out of 71 of the *T. macedonicus* localities for which sequence data are available, 35 have only original *T. macedonicus* mitochondrial DNA, 34 have only *T. karelinii* mitochondrial DNA and two localities show syntopy of both mitochondrial DNA types (Figure 2a). There is no *T. macedonicus* mitochondrial DNA found in any of the 86 *T. karelinii* localities for which sequence data is available. Based on the known geographical distribution of mitochondrial DNA we interpret mitochondrial DNA type for the remaining localities for which sequence data are not available (Figure 2a). By employing Thiessen polygons, we delimit an extensive (ca. 54,000 km^2^) area of asymmetric mitochondrial DNA introgression, in which *T. macedonicus* contains *T. karelinii* mitochondrial DNA (Figure 2b).

**Figure 3:**
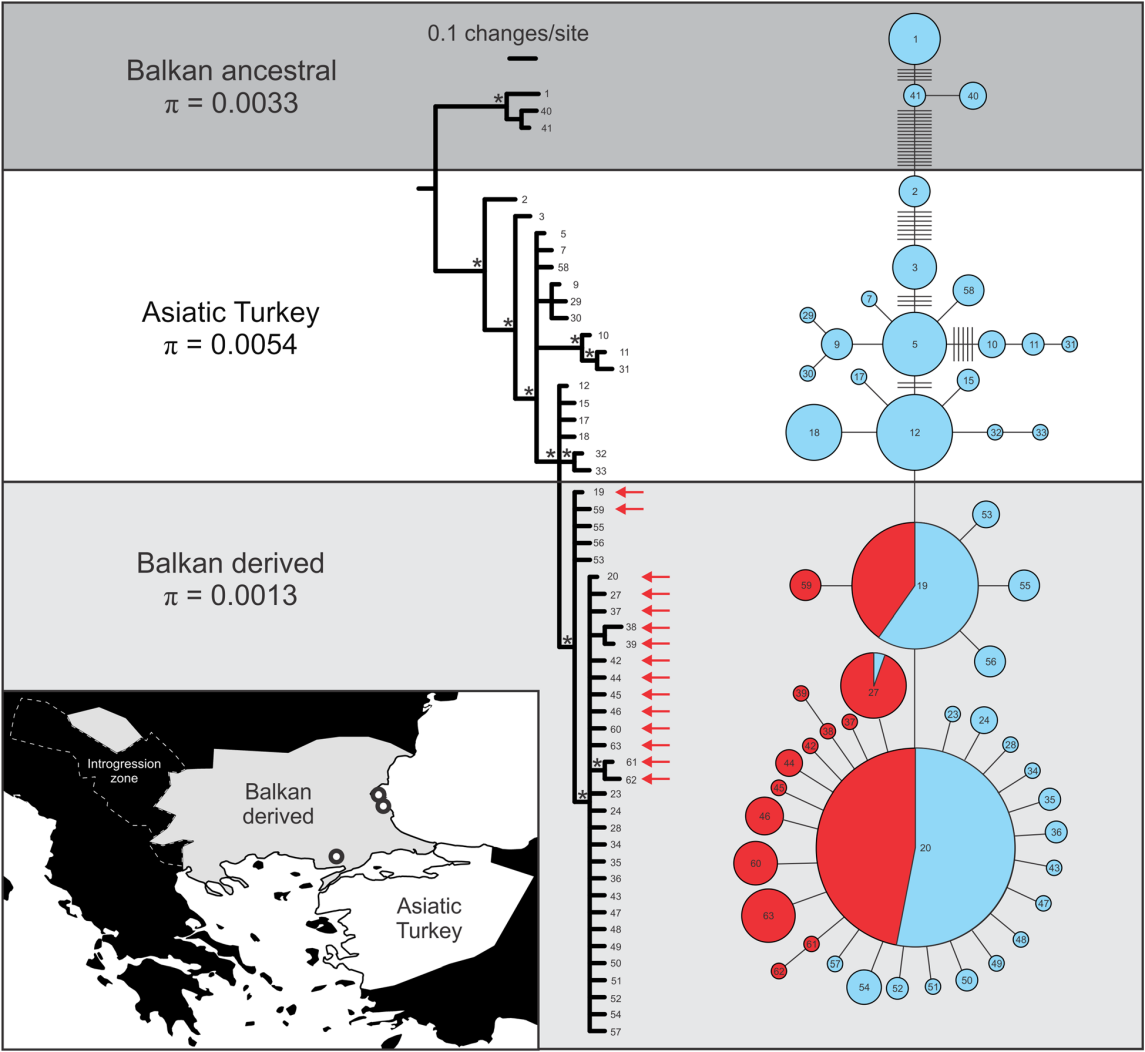
**The genetic structuring of *Triturus karelinii* mitochondrial DNA, including that introgressed into *T. macedonicus* .** Three groups of haplotypes are recognized: ‘Balkan ancestral’, ‘Asiatic Turkey’ and ‘Balkan derived’. Note that ‘Balkan derived’ is nested within ‘Asiatic Turkey’. The inset shows the geographical range of each group: ‘Asiatic Turkey’ in white and ‘Balkan derived’ in light grey (note the enclave); localities in which ‘Balkan ancestral’ haplotypes are found are marked with open circles (see Additional file 1 for details on haplotype distribution). The area where *T. macedonicus* contains *T. karelinii* mitochondrial DNA is outlined with a white stippled line. For each group the nucleotide diversity (π) is determined. In the phylogenetic tree, red arrows signify haplotypes that are (also) found as introgressed mitochondrial DNA in *T. macedonicus*. Statistically significantly supported clades (with a Bayesian posterior probability of 0.95 or higher) are denoted with an asterisk (*). In the haplotype network, pie diameter expresses haplotype frequency (Additional file 3) and bars the number of mutations along a branch if more than one. Blue pies reflect *T. karelinii* mitochondrial DNA found in *T. karelinii* and red pies *T. karelinii* mitochondrial DNA present in *T. macedonicus*. If *T. karelinii* haplotypes are shared between *T. karelinii* and *T. macedonicus*, frequencies are reflected by the size of the slices. The numbers in the phylogeny and haplotype network refer to *T. karelinii* mitochondrial DNA haplotypes as coded in Additional file 3.

**Figure 4:**
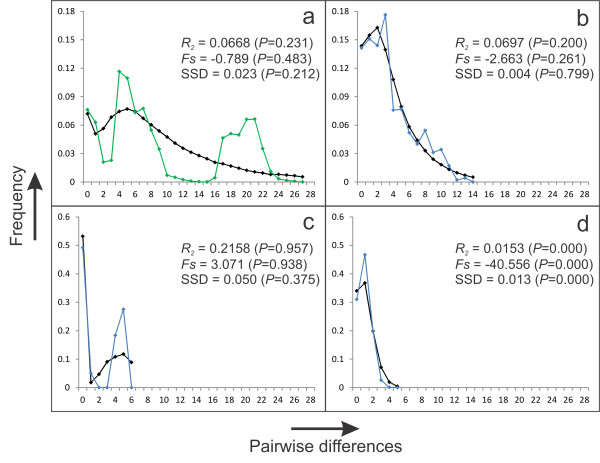
**Mismatch distributions for haplotype groups.** Groups of haplotypes are: *T. macedonicus* (**a**), *T. karelinii* ‘Asiatic Turkey’ (**b**), *T. karelinii* ‘Balkan ancestral’ (**c**) and *T. karelinii* ‘Balkan derived’ (**d**). The colored curves (green for *T. macedonicus* and blue for the three groups of *T. karelinii* haplotypes) show the observed frequency distribution of pairwise differences and the black curves the distribution that would be expected for an expanding population. Based on Ramos-Onsins and Rozas’ *R*_*2*_ and Fu’s *Fs* statistics, the null hypothesis of a constant population size is rejected for *T. karelinii* ‘Balkan derived’ only. This corresponds to the shape of the mismatch distributions: the unimodal and smooth distribution for *T. karelinii* ‘Balkan derived’ is indicative of demographic expansion. The sum of square deviations (SSD) rejecting the null model of population expansion for *T. karelinii* ‘Balkan derived’ may well reflect that the model of population expansion applied in Arlequin is overly simplistic.

The *T. karelinii* mitochondrial DNA shows extensive structuring and we discuss three (not necessarily monophyletic) spatio-temporal groups (Figure 3). A ‘Balkan ancestral’ clade, restricted to the extreme south-east Balkan Peninsula, is distinct from the remaining *T. karelinii* haplotypes (D_XY_ = 0.035). A genetically diverse group of haplotypes is distributed in western ‘Asiatic Turkey’ (Figure 3). A ‘Balkan derived’ clade is nested within, and closely related to, the ‘Asiatic Turkey’ haplotypes (separated by a single substitution). The ‘Balkan derived’ haplotypes show a starburst pattern: they are genetically similar, comprising a few common and a large number of rare haplotypes (Figure 3). For the *T. karelinii* mitochondrial DNA, only the ‘Balkan derived’ clade shows signs of demographic expansion (Figure 4). The original *T. macedonicus* mitochondrial DNA is genetically diverse (Additional file 2) and does not indicate demographic growth (Figure 4). All introgressed *T. karelinii* mitochondrial DNA belongs to the ‘Balkan derived’ clade (Figure 3). Three introgressed haplotypes are also found in *T. karelinii* (two of which represent the most frequent haplotypes found in both species) and 12 have only been identified in *T. macedonicus*.

**Figure 5:**
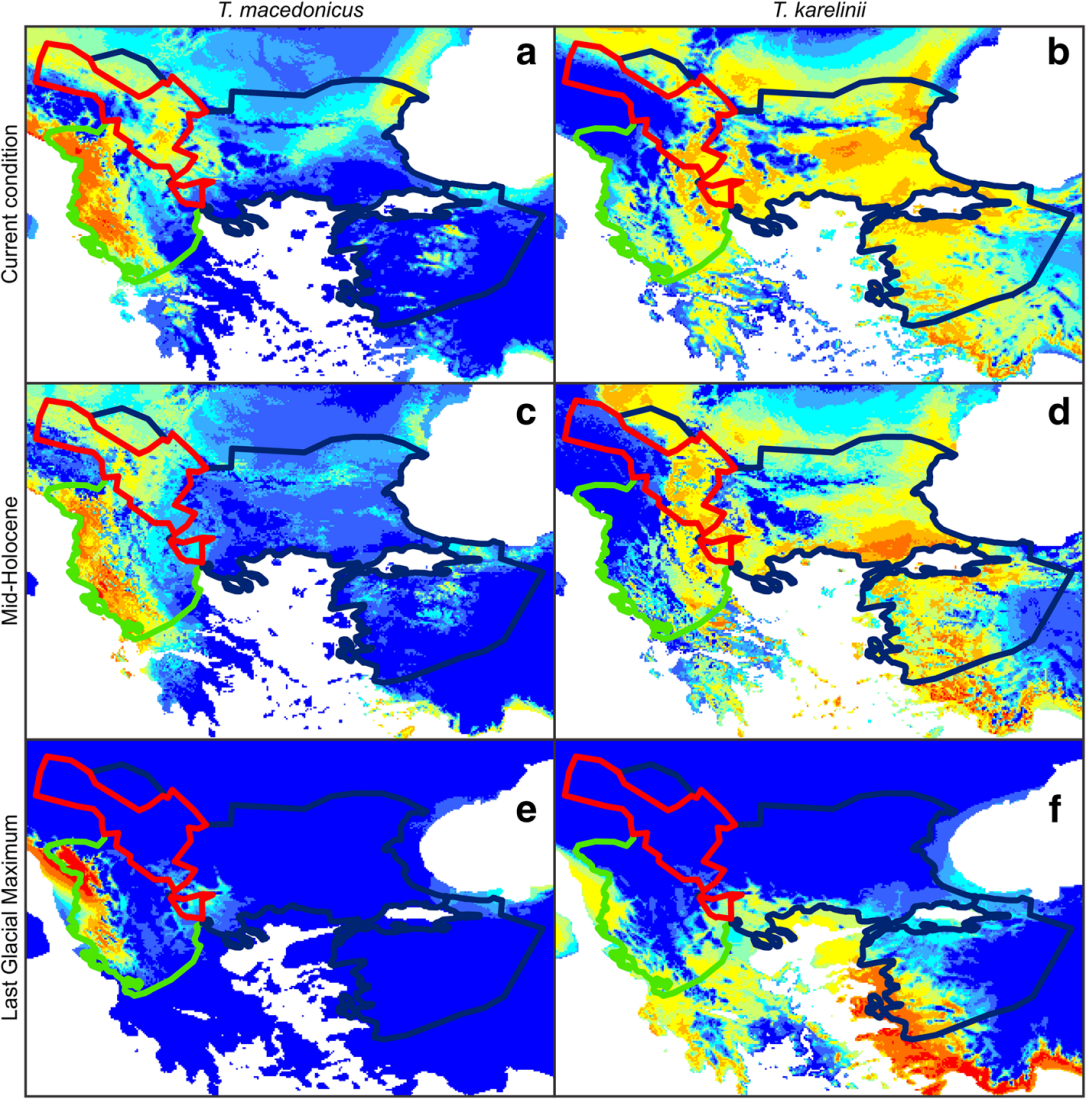
**The predicted distribution for *Triturus macedonicus* and *T. karelinii* under current and Last Glacial Maximum conditions based on ecological niche modeling.** The ecological niche models projected on current, mid-Holocene (~6Ka) and Last Glacial Maximum (~21Ka) climate layers for *T. macedonicus* are shown on the left (**a**, **c**, **e**) and those for *T. karelinii* on the right (**b**, **d**, **f**). The warmer a grid cell’s color, the higher its predicted suitability. The current range of *T. karelinii* is outlined in blue and that of *T. macedonicus* in green, with the zone of asymmetric mitochondrial DNA introgression outlined in red.

The ecological niche models of both crested newt species perform statistically significantly better than expected by random chance (Additional file 4). The ecological niche models are most affected by the mean temperature of the coldest quarter in both species and precipitation in the wettest quarter plays an important additional role in *T. macedonicus* (Additional file 5). When projected on Last Glacial Maximum (~21 Ka) data layers, the models suggest that the ranges of both crested newt species were restricted at the time; the present day introgression zone was uninhabitable (Figure 5). At the time of the mid-Holocene (~6 Ka) the two species would have been able to occupy much of their current range, but the predicted suitability maps differ from the current situation (Figure 5). The suitability of the area where the introgression zone is situated generally increased for *T. macedonicus* since the mid-Holocene, whereas it decreased for *T. karelinii*. The northwestern part of the current introgression zone is predicted to have been continuously unsuitable for *T. karelinii* (Figure 5).

## Discussion

### A scenario of species displacement

We confirm that the range of *T. karelinii* is bisected by *T. macedonicus* (Figure 2a), corroborating a previously hypothesized *T. karelinii* enclave [19]. Considering that crested newts are surface-bound and have limited dispersal capabilities, the presence of the *T. karelinii* enclave is best explained by it having been cut off from the main *T. karelinii* range by expansion of *T. macedonicus*. However, the mitochondrial DNA data suggest that a considerably larger area is involved in the geographical displacement of *T. karelinii* by *T. macedonicus* than that required to explain the enclave per se (Figure 2b). How was this introgression zone established? The structuring of the mitochondrial DNA of the two crested newt species and predicted distributions based on ecological niche modeling provide useful insight.

The ranges of both crested newt species were reduced at the Last Glacial Maximum (Figure 5). For *T. karelinii*, the southwestern margin of its current Balkan range (extending into land currently occupied by *T. macedonicus*) is predicted to have been hospitable. However, the mitochondrial DNA data suggest that the ‘Balkan ancestral’ clade was likely restricted to the south-east, whereas the ‘Balkan derived’ clade originates from a recent colonization from Asiatic Turkey (Figure 3). Indeed, Turkey’s west coast was also predicted suitable at the Last Glacial Maximum and mitochondrial DNA indicates a stable demographical history here (Figure 5). Furthermore, the ‘Balkan derived’ clade shows the signature of demographic growth, in line with a range expansion (Figure 4). The west side of the current range of *T. macedonicus* was also predicted to have been suitable at the Last Glacial Maximum, corresponding to long-lasting presence *in situ* as suggested by the mitochondrial DNA data (Figures 4 and 5).

The area where the introgression zone is situated was uninhabitable for both crested newt species at the Last Glacial Maximum and secondary contact between the two was established after glacial conditions had alleviated (Figure 5). Furthermore, the *T. karelinii* haplotypes introgressed into *T. macedonicus* are similar or identical to those found in *T. karelinii* itself. Taken together, this suggests that introgression happened after the Last Glacial Maximum. We did not detect more ancient mitochondrial DNA introgression (as found in other taxa by e.g. [22-25]). Given the climatic oscillations during the Quaternary Ice Age, the two newt species are presumed to have been in periodic contact through time during previous interglacials. However, as subsequent glacial periods would cause their ranges to recede again, any mitochondrial DNA that became introgressed in the area outside of their glacial refugia would be erased.

The climate changed considerably between the Last Glacial Maximum and the present, but we have an intervening snapshot at the mid-Holocene. Compared to the mid-Holocene, the suitability of the location of the present day introgression zone generally increased for *T. macedonicus*, whereas it decreased for *T. karelinii* (Figure 5). Life history differences between the two species provide some suggestion of how *T. macedonicus* was subsequently able to outcompete *T. karelinii*, under the changing abiotic environment. The two crested newt species appear to differ in the time annually allocated to an aquatic and a terrestrial life style, with adult *T. macedonicus* presumed to spend one more month in the water than *T. karelinii*[13,14]. This suggests that *T. macedonicus* is adapted to relatively wetter conditions. Bioclimatic variable contribution to the ecological niche models is in line with this hypothesis: the occurrence of *T. macedonicus* correlates with a higher precipitation than that of *T. karelinii* (Additional file 5). The climate layers used in the environmental niche modeling provide further insight: since the mid-Holocene, precipitation seasonality in the area where the introgression zone is positioned decreased; whereas precipitation during the wettest season decreased, it increased during the driest season (Additional file 6). Further research is required to understand the adaptive advantage of *T. macedonicus* over *T. karelinii* in the introgression zone.

**Figure 6:**
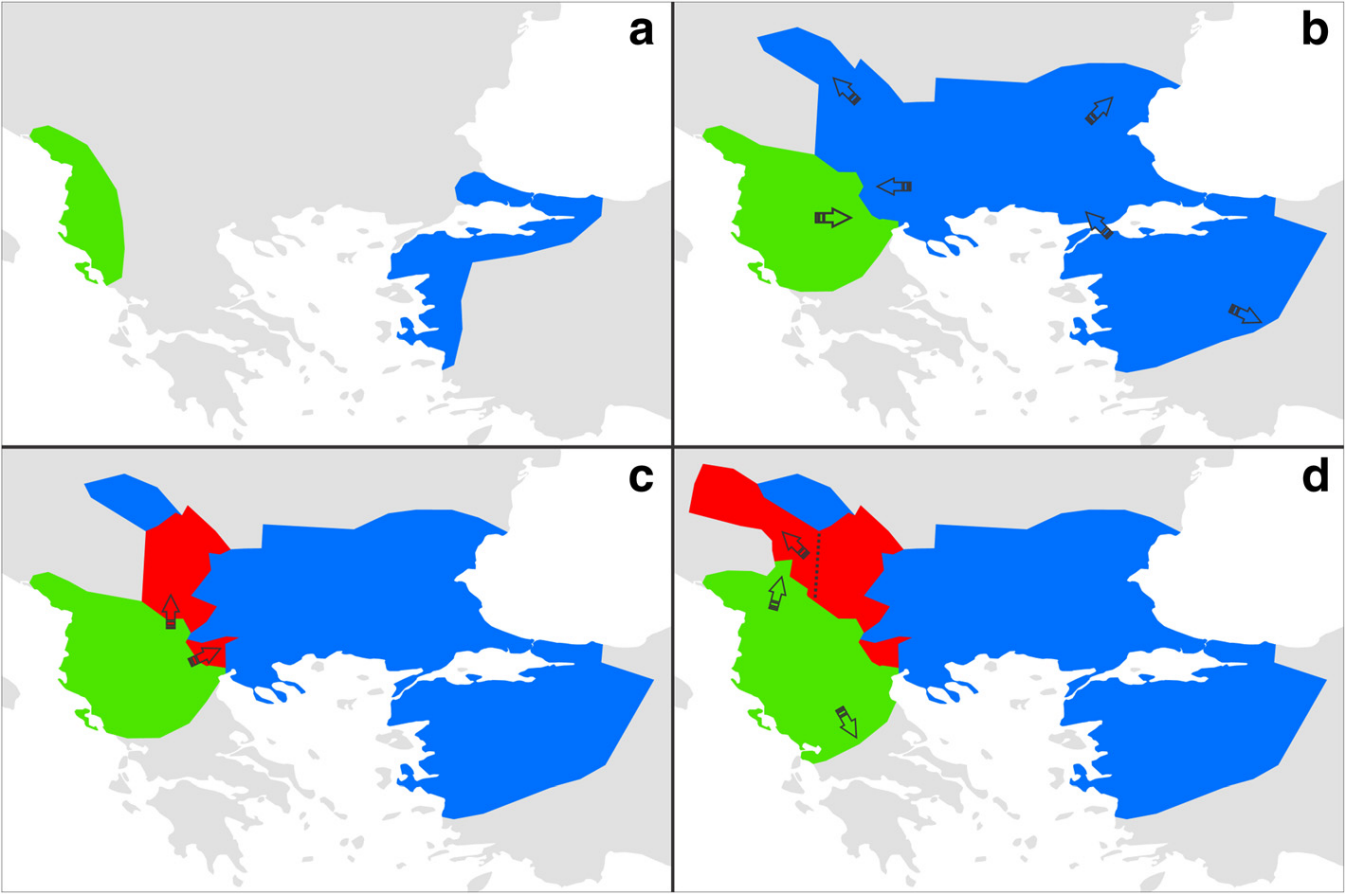
**A historical biogeographical scenario explaining the presence of *Triturus karelinii* mitochondrial DNA in *T. macedonicus* .** The ranges of *T. karelinii* and *T. macedonicus* are shown in blue and green and the region where *T. macedonicus* contains *T. karelinii* mitochondrial DNA is shown in red. During the last glaciation, both species’ ranges were repressed (**a**). After glacial conditions alleviated, *T. karelinii* derived from Turkey colonized a considerable part of the Balkan Peninsula and *T. macedonicus* expanded its range and came into contact with *T. karelinii* (**b**). Subsequently, *T. macedonicus* displaced *T. karelinii* over part of its range and in the process cut off a *T. karelinii* enclave; due to mitochondrial DNA introgression, *T. macedonicus* possessed *T. karelinii* mitochondrial DNA, there where it displaced *T. karelinii* (**c**). Finally, *T. macedonicus* expanded its range further and, as part of the source population contained *T. karelinii* mitochondrial DNA, this mitochondrial DNA spread, via the bodies of *T. macedonicus*, into an area not inhabited by *T. karelinii* itself (**d**).

For *T. karelinii*, the northwestern part of the current introgression zone is predicted to be unsuitable currently, but also in the two past time periods (Figure 5). Note that *T. karelinii* probably never colonized this part of the introgression zone: *T. karelinii* mitochondrial DNA probably reached this region via its *T. macedonicus* host. The reasoning is as follows: *T. macedonicus* newts that colonized the northwestern part of the introgression zone derived from the stock in the south of the introgression zone. As this *T. macedonicus* stock already possessed *T. karelinii* mitochondrial DNA, no matter if there were *T. karelinii* present in the northwestern part of the introgression zone, *T. macedonicus* would spread *T. karelinii* mitochondrial DNA here, not *T. macedonicus* mitochondrial DNA. We summarize the hypothesized distribution dynamics of the two crested newt species in Figure 6.

### Reinforcement of asymmetric mitochondrial DNA transmission

Geographical species displacement involves a demographical inequality: at the leading edge, the invader is rare whereas the species being displaced is common. This demographic inequality is in itself sufficient to explain asymmetrical mitochondrial DNA introgression [4]. However, the pattern can be reinforced by male biased dispersal and through the operation of Haldane’s rule [26]. Males dispersing into territory occupied by a species with which they can hybridize would only encounter females of the indigenous species to mate with. Most of their genetic material can potentially be transmitted to future generations, but their offspring would always contain indigenous mitochondrial DNA, as this is transmitted matrilinearily only. Whether there is male-biased dispersal in crested newts is not known. Haldane’s rule [27] states that if one sex is absent, rare, or sterile in F1 offspring, that sex is the heterogametic sex. In the case of crested newts, this would be the male [28]. In effect, markers transmitted via the female line (i.e. mitochondrial DNA) would be particularly prone to introgress [26]. Male biased dispersal and Haldane’s rule could act in combination while assortative mating would further amplify asymmetric mitochondrial DNA introgression [4]: if members of the native species prefer to mate among themselves, the invading males and the excess of hybrid females would be more likely to mate with each other.

Even when the parental species occurs in the same frequency, an asymmetry in mitochondrial DNA transmission could be strengthened by factors favoring the relative frequency (prezygotic factors) or viability (postzygotic factors) of one hybrid class over the other [2-4]. Examples of pre- and postzygotic selection are known in nature. In the tree frogs *Hyla cinerea* and *H. gratiosa*, differences in behavior (courting at the edge versus inside the breeding pond and presence versus absence of satellite mating behavior) cause *H. cinerea* males to more often intercept *H. gratiosa* females than vice versa, resulting in biased mitochondrial DNA transmission [29]. For the crested newt *T. cristatus* and the marbled newt *T. marmoratus*, F1 hybrids make up about four percent of the adult population where the ranges of the two species overlap [30]. Uneven viability of hybrids causes the frequency of *T. marmoratus*-mothered hybrids to drop from fifty percent in embryos to ten percent in adults. The *T. cristatus*-mothered hybrids show an excess of females (i.e. a Haldane’s effect). However, the *T. marmoratus*-mothered hybrids consist of males only, suggesting an incompatibility between the *T. cristatus* X chromosome and *T. marmoratus* cytoplasm [30].

In the present case, a higher frequency or reproductive success of *T. karelinii*-mothered hybrids over *T. macedonicus*-mothered hybrids would have acted like a filter, hampering the spread of *T. macedonicus* mitochondrial DNA into the introgression zone. Research into the presence of such asymmetries in *T. macedonicus* – *T. karelinii* hybridization is a prospect for future study.

### What about positive selection?

Asymmetric mitochondrial DNA introgression due to the positive selection of foreign mitochondrial DNA [6,8,31] is characterized by a distinctly different pattern of geographical spread than if caused by species displacement: instead of the contact zone between two species moving across the initial mitochondrial DNA boundary, which is the case with geographical displacement (Figure 1b), positive selection results in mitochondrial DNA of one species being ‘pulled’ into the range of the other. A selective sweep, where foreign mitochondrial DNA replaces the original mitochondrial DNA, has been demonstrated experimentally in *Drosophila* flies [32]. Positive selection has also been suggested to cause asymmetric mitochondrial DNA introgression in nature [33,34]. However, when encountering asymmetric mitochondrial DNA introgression, it is difficult to choose between a scenario of species displacement or natural selection. For example, in *Lissotriton* newts the original mitochondrial DNA of *L. montandoni* has been fully replaced by that of *L. vulgaris*[35]. However, this exchange of mitochondrial DNA did not have a single origin but involved multiple, independent introgression events of well-differentiated *L. vulgaris* mitochondrial DNA. The observed pattern could either reflect repeated geographical replacement during multiple interglacials, when the more mountainous *L. montandoni* shifted its range to a lower elevation, or several uptakes of foreign mitochondrial DNA that subsequently spread in *L. montandoni* by natural selection.

Why do we in the present case favor the geographical displacement scenario over the positive selection scenario? The bisecting of the *T. karelinii* range by *T. macedonicus* provides positive evidence in favor of *T. macedonicus* expanding its range at the expense of *T. karelinii*. This displacement is further supported by *T. karelinii* mitochondrial DNA having spread into a region where we infer *T. karelinii* was never present – this would require a *T. macedonicus* host that was expanding its range after having captured *T. karelinii* mitochondrial DNA. Furthermore, foreign *T. karelinii* mitochondrial DNA is only present in *T. macedonicus* in postglacially colonized areas. If positive selection had caused the asymmetric mitochondrial DNA introgression, foreign mitochondrial could also have penetrated into the region that acted as a glacial refugium for *T. macedonicus*. Only if an adaptive advantage of foreign mitochondrial DNA was somehow limited to the area where the introgression zone is currently positioned, it would be indistinguishable from a scenario involving species displacement. However, we know that *T. macedonicus* has at least partially displaced *T. karelinii*, based on the well-supported *T. karelinii* enclave. Considering that species displacement occurred at least in part of the introgression zone, the most parsimonious explanation is that species displacement alone caused the pattern of asymmetric mitochondrial DNA introgression observed, instead of a combination of species displacement and localized positive selection of mitochondrial DNA. To test this, future research should employ a large battery of nuclear DNA sequences. Selectively neutral nuclear genes would be expected to show a comparable geographical pattern of introgression compared to mitochondrial DNA when asymmetrical mitochondrial DNA introgression was caused by species displacement [4]. This would, however, not be the expectation in the case of positive selection of mitochondrial DNA.

## Conclusions

The *T. macedonicus* – *T. karelinii* case provides a unique insight into asymmetric mitochondrial DNA introgression. The fact that *T. macedonicus* bisects the range of *T. karelinii* provides a clear indication of directionality: *T. macedonicus* likely invaded *T. karelinii* territory instead of the other way around. Based on a combination of phylogeography and ecological niche modeling, we suggest that the asymmetric introgression of mitochondrial DNA from *T. karelinii* into *T. macedonicus* is due to postglacial species displacement (see the summarized scenario in Figure 6). As these crested newt species expanded their ranges in response to climate change after the conclusion of the Last Glacial Maximum, they came into spatial contact. Subsequently, the newly established hybrid zone between the two moved across the landscape, as *T. macedonicus* outcompeted *T. karelinii*. However, the former distribution of *T. karelinii*, before *T. macedonicus* started to invade its range, could be inferred based on the asymmetrically introgressed *T. karelinii* mitochondrial DNA. The asymmetrical introgression of mitochondrial DNA has been regularly documented in a wide range of taxa and can provide key insights into historical biogeography [4,36].

## Methods

### Distribution and genetic data

We included 139 *T. macedonicus* and 135 *T. karelinii* localities (Figure 2 and Additional file 1). Species identity was primarily based on morphological characters (throat and belly pattern and morphological measurements); for a subset (conveniently arranged along transects across the contact zone) diagnostic allozyme markers have been published [17,18], Arntzen et al. submitted]. Note that at five localities the species are found in syntopy. We obtained a 658-bp segment of ND4 for 600 newts (70 previously published in, and 530 newly sequenced following the protocol of [12]) from 71 of the *T. macedonicus* and 86 of the *T. karelinii* localities (Figure 2 and Additional file 1). Among them, the sequenced individuals contained 83 haplotypes (Additional file 3). We delimited the zone of asymmetrically introgressed mitochondrial DNA by Thiessen polygons, using ArcGIS (http://www.esri.com). Each polygon represents a locality and contains the area that is closer to that particular locality than to another one [as in [37].

### Genetic analyses

Phylogenetic trees were constructed with MrBayes 3.1.2 [38], employing two, four-chain, twenty million generation runs, with a sampling frequency of 0.001 and a heating parameter of 0.1. MrModeltest [39] identified GTR + I, HKY + I + G and HKY + G as the most suitable models of sequence evolution for codon positions 1, 2 and 3. Data partitions were unlinked. Tracer 1.5 [40] was used to check for stabilization of overall likelihood within and convergence between runs. The first quarter of the sampled trees was discarded as burn-in. As an outgroup we used *T. marmoratus* (GenBank accession number GU982379). Minimum spanning haplotype networks were created with HapStar 0.5 [41], based on distance matrices produced with Arlequin 3.5 [42]. DnaSP5 [43] was used to determine the nucleotide diversity (π) within and the average number of nucleotide substitutions (D_XY_) between groups of haplotypes. To test for signals of demographic expansion, we compared the observed mismatch distribution with that expected under population expansion [44]. We interpreted the differences based on the Ramos-Onsins and Rozas’ *R*_*2*_ and Fu’s *Fs* statistics [45], for which statistical significance was obtained with a 1,000 coalescent simulations in DNAsp, and based on the sum of square deviations, for which statistical was evaluated with a 1,000 bootstrap replicates, in Arlequin 3.5 [42].

### Environmental data

For climate layers we used bioclimatic variables at 2.5 arcminute resolution, available from WorldClim 1.4 [46]. To obtain realistic and transferable ecological niche models, it is recommended to mirror the physiological limitations of the study species and minimize the effects of multi-colinearity among data layers [47-50]. Crested newt species differ in the length of their annual aquatic period [13,14]. Therefore, we include a set of layers that likely reflects the availability of water bodies during the breeding season, i.e. seasonal variation in evaporation and precipitation: bio10 = mean temperature of warmest quarter, bio11 = mean temperature of coldest quarter, bio15 = precipitation seasonality, bio16 = precipitation of wettest quarter, and bio17 = precipitation of driest quarter. These layers show a Pearson correlation < 0.7. Bioclimatic variables are also available for the mid-Holocene (~6 Ka) and the Last Glacial Maximum (~21 Ka), being derived from the Paleoclimate Modelling Intercomparison Project phase 2 [51]; http://pmip2.lsce.ipsl.fr/. We used datalayers for the former based on simulations using the ECHAM3 model (Model Max Planck Inst. für Meteorologie) and the latter based on simulations using the Community Climate System Model version 3 (CCSM) [52].

### Ecological niche modeling

Ecological niche models were created using Maxent 3.3.3e [53]. We restricted the feature type to hinge features as this produces smoother model fits, so forcing models to be more focused on key trends rather than potential idiosyncrasy in the data, which would hamper extrapolation to a different time or place [54]. We restricted the area from which pseudo-absence was drawn to the distribution of the entire crested newt *T. cristatus* superspecies. This area was broadly defined as a 200 km buffer zone [55] around known crested newt localities [Wielstra et al., submitted]. The ecological niche models were tested for statistical significance against a null model derived from random localities, to test whether they perform better than expected by random chance [56]. We created a null distribution of 99 AUC values, based on as many random localities as used for the tested species distribution model. The AUC value of the tested species distribution model was treated as a 100^th^ value and deemed statistically significant if it ranked higher than the 95^th^ value (i.e. above the 95% confidence interval). Random point data were created with ENMTools 1.3 [57]; http://enmtools.blogspot.com/. The null model approach prevents interpreting model quality based on an arbitrary AUC threshold and precludes the requirement to set aside part of the localities for model testing [56]. Models were projected on the current and Last Glacial Maximum climate layers. We explored variable importance and response curves and conducted a jackknife test to determine how ecological niche models of the two species differed.

## Competing interests

The authors declare that they have no competing interests.

## Authors’ contributions

BW conducted laboratory work and analyzed the data. BW and JWA designed research and wrote the paper. Both authors read and approved the final manuscript.

## Supplementary Material

Additional file 1 **Sampling.***Triturus karelinii* and *T. macedonicus* localities included in the genetic analysis (a subset) and in ecological niche modelling (all). Click here for file

Additional file 2 ** *Triturus macedonicus* mitochondrial DNA structuring.** A phylogenetic tree and haplotype network for *T. macedonicus* and a spatial visualization of the distribution of groups of haplotypes. Click here for file

Additional file 3 **List of mitochondrial DNA haplotypes.** For each mitochondrial DNA haplotype, GenBank accession number and frequency of occurrence in both* Triturus macedonicus and T. karelinii is provided.* tested against a null model. Click here for file

Additional file 4 **Ecological niche modeling performance.** The ecological niche models for *Triturus macedonicus* and *T. karelinii* tested against a null model. Click here for file

Additional file 5 **Contribution of bioclimatic variables to the ecological niche models.** Response curves, contribution and permutation importance and results of a jackknife test for the bioclimatic variables. Click here for file

Additional file 6 **Differences between the current and mid-Holocene climate layers.** For each of the bioclimatic values used in the ecological niche modeling, cells with a higher value under current climate compared to the mid-Holocene are shown in red and cells with a lower value in blue. Click here for file

## References

[B1] Coyne JA Orr HA Speciation 2004 Sinauer Associates, Sunderland

[B2] Chan KMA Levin SA Leaky prezygotic isolation and porous genomes: Rapid introgression of maternally inherited DNA Evolution 2005 59 4 720 729 15926684

[B3] Excoffier L Foll M Petit RJ Genetic consequences of range expansions Annu Rev Ecol Evol Syst 2009 40 481 501 10.1146/annurev.ecolsys.39.110707.173414

[B4] Currat M Ruedi M Petit RJ Excoffier L The hidden side of invasions: Massive introgression by local genes Evolution 2008 62 8 1908 1920 18452573 10.1111/j.1558-5646.2008.00413.x

[B5] Petit RJ Excoffier L Gene flow and species delimitation Trends Ecol Evol 2009 24 7 386 393 10.1016/j.tree.2009.02.011 19409650

[B6] Galtier N Nabholz B Glemin S Hurst GDD Mitochondrial DNA as a marker of molecular diversity: A reappraisal Mol Ecol 2009 18 22 4541 4550 10.1111/j.1365-294X.2009.04380.x 19821901

[B7] Gyllensten U Wharton D Wilson AC Maternal inheritance of mitochondrial DNA during backcrossing of two species of mice J Hered 1985 76 5 321 324 2997326 10.1093/oxfordjournals.jhered.a110103

[B8] Ballard JWO Whitlock MC The incomplete natural history of mitochondria Mol Ecol 2004 13 4 729 744 10.1046/j.1365-294X.2003.02063.x 15012752

[B9] Hewitt G The genetic legacy of the Quaternary ice ages Nature 2000 405 6789 907 913 10.1038/35016000 10879524

[B10] Kozak KH Graham CH Wiens JJ Integrating GIS-based environmental data into evolutionary biology Trends Ecol Evol 2008 23 3 141 148 10.1016/j.tree.2008.02.001 18291557

[B11] Svenning J-C Fløjgaard C Marske KA Nógues-Bravo D Normand S Applications of species distribution modeling to paleobiology Quaternary Sci Rev 2011 30 21–22 2930 2947

[B12] Wielstra B Espregueira Themudo G Güclü Ö Olgun K Poyarkov NA Arntzen JW Cryptic crested newt diversity at the Eurasian transition: The mitochondrial DNA phylogeography of Near Eastern Triturus newts Mol Phylogenet Evol 2010 56 3 888 896 10.1016/j.ympev.2010.04.030 20435147

[B13] Wielstra B Arntzen JW Unraveling the rapid radiation of crested newts (Triturus cristatus superspecies) using complete mitogenomic sequences BMC Evol Biol 2011 11 162 10.1186/1471-2148-11-162 21672214 PMC3224112

[B14] Arntzen JW Grossenbacher K, Thiesmeier B Triturus cristatus Superspecies - Kammolch-Artenkreis (Triturus cristatus (Laurenti, 1768) - Nördlicher Kammolch, Triturus carnifex (Laurenti, 1768) - Italienischer Kammolch, Triturus dobrogicus (Kiritzescu, 1903) - Donau-Kammolch, Triturus karelinii (Strauch, 1870) - Südlicher Kammolch) Handbuch der Reptilien und Amphibien Europas Schwanzlurche IIA 2003 Aula-Verlag, Wiebelsheim 421 514

[B15] Espregueira Themudo G Wielstra B Arntzen JW Multiple nuclear and mitochondrial genes resolve the branching order of a rapid radiation of crested newts (Triturus, Salamandridae) Mol Phylogenet Evol 2009 52 2 321 328 10.1016/j.ympev.2009.03.024 19348957

[B16] Wiens JJ Sparreboom M Arntzen JW Crest evolution in newts: Implications for reconstruction methods, sexual selection, phenotypic plasticity and the origin of novelties J Evol Biol 2011 24 10 2073 2086 10.1111/j.1420-9101.2011.02340.x 21707814

[B17] Arntzen JW Wielstra B Where to draw the line? A nuclear genetic perspective on proposed range boundaries of the crested newts Triturus karelinii and T. arntzeni Amphibia-Reptilia 2010 31 3 311 322 10.1163/156853810791769509

[B18] Arntzen JW Genetic variation in the Italian crested newt, Triturus carnifex, and the origin of a non-native population north of the Alps Biodivers Conserv 2001 10 6 971 987 10.1023/A:1016644814551

[B19] Arntzen JW Wallis GP Geographic variation and taxonomy of crested newts (Triturus cristatus superspecies): Morphological and mitochondrial data Contrib Zool 1999 68 3 181 203

[B20] Wallis GP Arntzen JW Mitochondrial-DNA variation in the crested newt superspecies: Limited cytoplasmic gene flow among species Evolution 1989 43 1 88 104 10.2307/2409166 28568488

[B21] Arntzen JW Espregueira Themudo G Wielstra B The phylogeny of crested newts (Triturus cristatus superspecies): Nuclear and mitochondrial genetic characters suggest a hard polytomy, in line with the paleogeography of the centre of origin Contrib Zool 2007 76 4 261 278

[B22] McGuire JA Linkem CW Koo MS Hutchison DW Lappin AK Orange DI Lemos-Espinal J Riddle BR Jaeger JR Mitochondrial introgression and incomplete lineage sorting through space and time: Phylogenetics of crotaphytid lizards Evolution 2007 61 12 2879 2897 10.1111/j.1558-5646.2007.00239.x 17941840

[B23] Bryson JRW De Oca AN-M Jaeger JR Riddle BR Elucidation of cryptic diversity in a widespread Nearctic treefrog reveals episodes of mitochondrial gene capture as frogs diversified across a dynamic landscape Evolution 2010 64 8 2315 2330 20394664 10.1111/j.1558-5646.2010.01014.x

[B24] Bossu CM Near TJ Gene trees reveal repeated instances of mitochondrial DNA introgression in orangethroat darters (Percidae: Etheostoma) Syst Biol 2009 58 1 114 129 10.1093/sysbio/syp014 20525572

[B25] Liu K Wang F Chen W Tu L Min M-S Bi K Fu J Rampant historical mitochondrial genome introgression between two species of green pond frogs, Pelophylax nigromaculatus and P. plancyi BMC Evol Biol 2010 10 1 201 10.1186/1471-2148-10-201 20587049 PMC2909235

[B26] Beysard M Perrin N Jaarola M Heckel G Vogel P Asymmetric and differential gene introgression at a contact zone between two highly divergent lineages of field voles (Microtus agrestis) J Evol Biol 2012 25 2 400 408 10.1111/j.1420-9101.2011.02432.x 22150868

[B27] Haldane JBS Sex ratio and unisexual sterility in hybrid animals J Genet 1922 12 2 101 109 10.1007/BF02983075

[B28] Macgregor HC An introduction to animal cytogenetics 1993 Chapman & Hall, London

[B29] Lamb T Avise JC Directional introgression of mitochondrial DNA in a hybrid population of tree frogs: The influence of mating behavior Proc Natl Acad Sci USA 1986 83 8 2526 2530 10.1073/pnas.83.8.2526 16593687 PMC323331

[B30] Arntzen JW Jehle R Bardakci F Burke T Wallis GP Asymmetric viability of reciprocal-cross hybrids between crested and marbled newts (Triturus cristatus and T. marmoratus) Evolution 2009 63 5 1191 1202 10.1111/j.1558-5646.2009.00611.x 19154385

[B31] Rand DM The units of selection on mitochondrial DNA Annu Rev Ecol Syst 2001 32 415 448 10.1146/annurev.ecolsys.32.081501.114109

[B32] Niki Y Chigusa SI Matsuura ET Complete replacement of mitochondrial DNA in Drosophila Nature 1989 341 6242 551 552 10.1038/341551a0 2507929

[B33] Irwin DE Rubtsov AS Panov EN Mitochondrial introgression and replacement between yellowhammers (Emberiza citrinella) and pine buntings (Emberiza leucocephalos) (Aves: Passeriformes) Biol J Linn Soc 2009 98 2 422 438 10.1111/j.1095-8312.2009.01282.x

[B34] Plötner J Uzzell T Beerli P Spolsky C Ohst T Litvinchuk SN Guex GD Reyer HU Hotz H Widespread unidirectional transfer of mitochondrial DNA: a case in western Palaearctic water frogs J Evol Biol 2008 21 3 668 681 10.1111/j.1420-9101.2008.01527.x 18373588 PMC2505272

[B35] Babik W Branicki W Crnobrnja-Isailovic J Cogalniceanu D Sas I Olgun K Poyarkov NA Garcia-Paris M Arntzen JW Phylogeography of two European newt species - Discordance between mtDNA and morphology Mol Ecol 2005 14 8 2475 2491 10.1111/j.1365-294X.2005.02605.x 15969729

[B36] Toews DPL Brelsford A The biogeography of mitochondrial and nuclear discordance in animals Mol Ecol 2012 21 16 3907 3930 10.1111/j.1365-294X.2012.05664.x 22738314

[B37] Arntzen JW Wallis GP Restricted gene flow in a moving hybrid zone of the newts Triturus cristatus and T. marmoratus in western France Evolution 1991 45 4 805 826 10.2307/2409691 28564049

[B38] Ronquist F Huelsenbeck JP MrBayes 3: Bayesian phylogenetic inference under mixed models Bioinformatics 2003 19 12 1572 1574 10.1093/bioinformatics/btg180 12912839

[B39] Nylander JAA MrModelTest2. Computer program distributed by the author Evolutionary Biology Centre 2004 Uppsala University, Sweden

[B40] Rambaut A Drummond AJ Tracer v1.4 2007, http://beast.bio.ed.ac.uk/Tracer

[B41] Teacher AGF Griffiths DJ HapStar: Automated haplotype network layout and visualization Mol Ecol Resour 2011 11 1 151 153 10.1111/j.1755-0998.2010.02890.x 21429113

[B42] Excoffier L Lischer HEL Arlequin suite ver 3.5: A new series of programs to perform population genetics analyses under Linux and Windows Mol Ecol Resour 2010 10 3 564 567 10.1111/j.1755-0998.2010.02847.x 21565059

[B43] Librado P Rozas J DnaSP v5: A software for comprehensive analysis of DNA polymorphism data Bioinformatics 2009 25 11 1451 1452 10.1093/bioinformatics/btp187 19346325

[B44] Rogers AR Harpending H Population growth makes waves in the distribution of pairwise genetic differences Mol Biol Evol 1992 9 3 552 569 1316531 10.1093/oxfordjournals.molbev.a040727

[B45] Ramos-Onsins SE Rozas J Statistical properties of bew beutrality tests against population growth Mol Biol Evol 2002 19 12 2092 2100 10.1093/oxfordjournals.molbev.a004034 12446801

[B46] Hijmans RJ Cameron SE Parra JL Jones PG Jarvis A Very high resolution interpolated climate surfaces for global land areas Int J Climatol 2005 25 15 1965 1978 10.1002/joc.1276

[B47] Rödder D Schmidtlein S Veith M Lötters S Alien invasive slider turtle in unpredicted habitat: A matter of niche shift or of predictors studied? PLoS One 2009 4 11 e7843 10.1371/journal.pone.0007843 19956684 PMC2776975

[B48] Austin M Species distribution models and ecological theory: A critical assessment and some possible new approaches Ecol Model 2007 200 1–2 1 19

[B49] Guisan A Thuiller W Predicting species distribution: Offering more than simple habitat models Ecol Lett 2005 8 9 993 1009 10.1111/j.1461-0248.2005.00792.x 34517687

[B50] Peterson AT Ecological niche conservatism: a time-structured review of evidence J Biogeogr 2011 38 5 817 827 10.1111/j.1365-2699.2010.02456.x

[B51] Braconnot P Otto-Bliesner B Harrison S Joussaume S Peterchmitt JY Abe-Ouchi A Crucifix M Driesschaert E Fichefet T Hewitt CD Results of PMIP2 coupled simulations of the Mid-Holocene and Last Glacial Maximum - Part 1: experiments and large-scale features Clim Past 2007 3 2 261 277 10.5194/cp-3-261-2007

[B52] Collins WD Bitz CM Blackmon ML Bonan GB Bretherton CS Carton JA Chang P Doney SC Hack JJ Henderson TB The Community Climate System Model Version 3 (CCSM3) J Climate 2006 19 11 2122 2143 10.1175/JCLI3761.1

[B53] Phillips SJ Anderson RP Schapire RE Maximum entropy modeling of species geographic distributions Ecol Model 2006 190 3–4 231 259

[B54] Elith J Kearney M Phillips S The art of modelling range-shifting species Methods Ecol Evol 2010 1 4 330 342 10.1111/j.2041-210X.2010.00036.x

[B55] VanDerWal J Shoo LP Graham C Williams SE Selecting pseudo-absence data for presence-only distribution modeling: How far should you stray from what you know? Ecol Model 2009 220 4 589 594 10.1016/j.ecolmodel.2008.11.010

[B56] Raes N ter Steege H A null-model for significance testing of presence-only species distribution models Ecography 2007 30 5 727 736 10.1111/j.2007.0906-7590.05041.x

[B57] Warren DL Glor RE Turelli M ENMTools: A toolbox for comparative studies of environmental niche models Ecography 2010 33 3 607 611

