# Supplementary material for: Postglacial species displacement in Triturus newts deduced from asymmetrically introgressed mitochondrial DNA and ecological niche models
Source: BMC Evol Biol. 2012 Aug 30;12:161. doi: 10.1186/1471-2148-12-161 (PMC3520116; doi:10.1186/1471-2148-12-161)
Supplement: Additional file 2 — Triturus macedonicus mitochondrial DNA structuring. A phylogenetic tree and haplotype network for T. macedonicus and a spatial visualization of the distribution of groups of haplotypes. [file 1471-2148-12-161-S2.pdf]

Additional file 2: *Triturus macedonicus* mitochondrial DNA structuring. A phylogenetic tree and haplotype network for *T. macedonicus* and a spatial visualization of the distribution of groups of haplotypes.

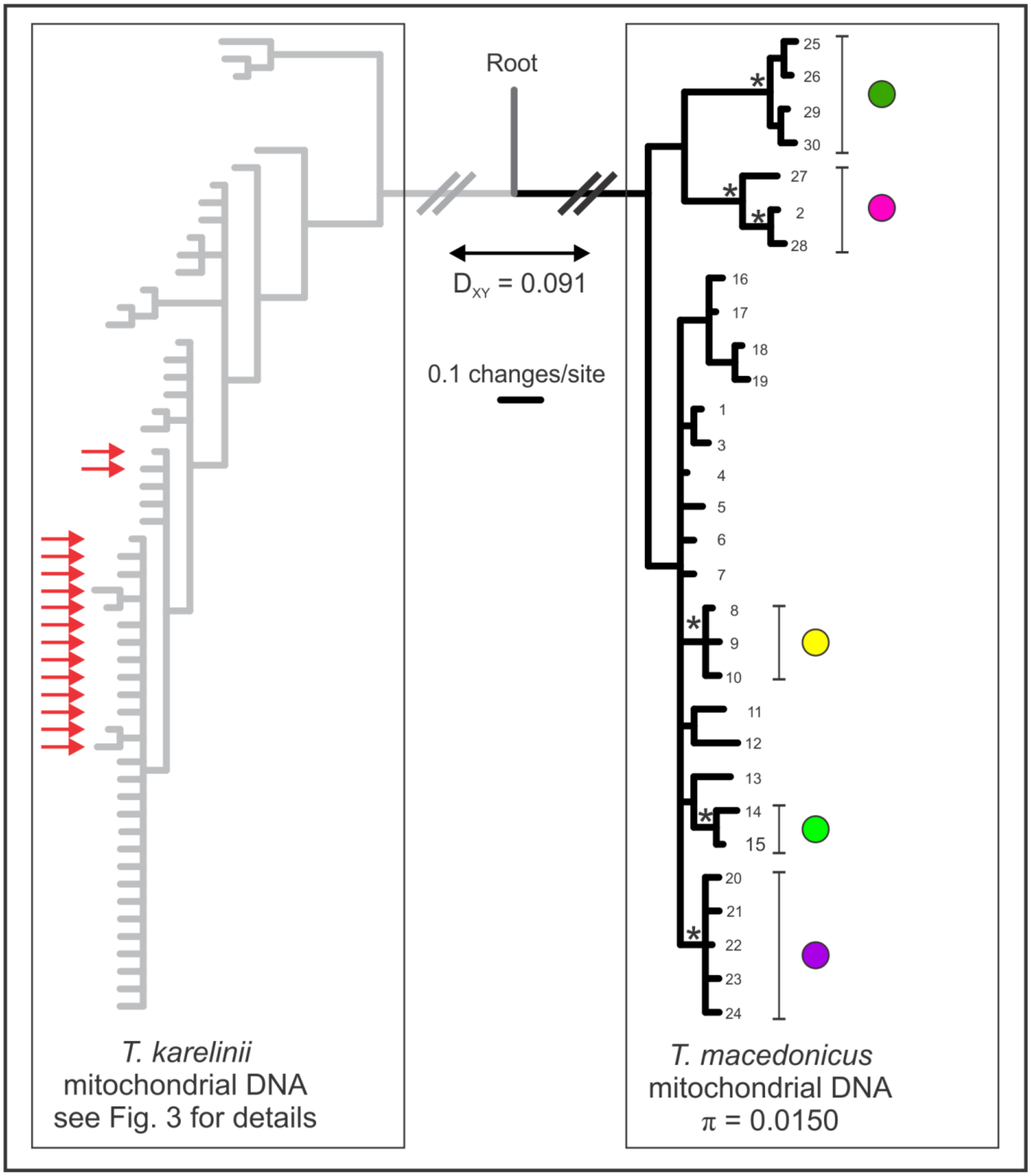

The phylogenetic tree on the right represents *T. macedonicus* mitochondrial DNA. Statistically significantly supported clades (with a Bayesian posterior probability of 0.95 or higher) are denoted with an asterisk (*).The numbers in the phylogeny refer to *T. macedonicus* mitochondrial DNA haplotypes as coded in Additional file 5. Colors correspond to the haplotype network and the map below; haplotypes for which no interspecific structuring is revealed are unmarked in the phylogeny and marked black in the haplotype netwok and the map below. The phylogeny on the left represents *T. karelinii* mitochondrial DNA and is a simplified version of Fig. 3. Note that the *T. karelinii* mitochondrial DNA is highly distinct from *T. macedonicus* mitochondrial DNA (D_{XY} = 0.091). The red arrows denote the *T. karelinii* mitochondrial DNA haplotypes that have introgressed into *T. macedonicus*.

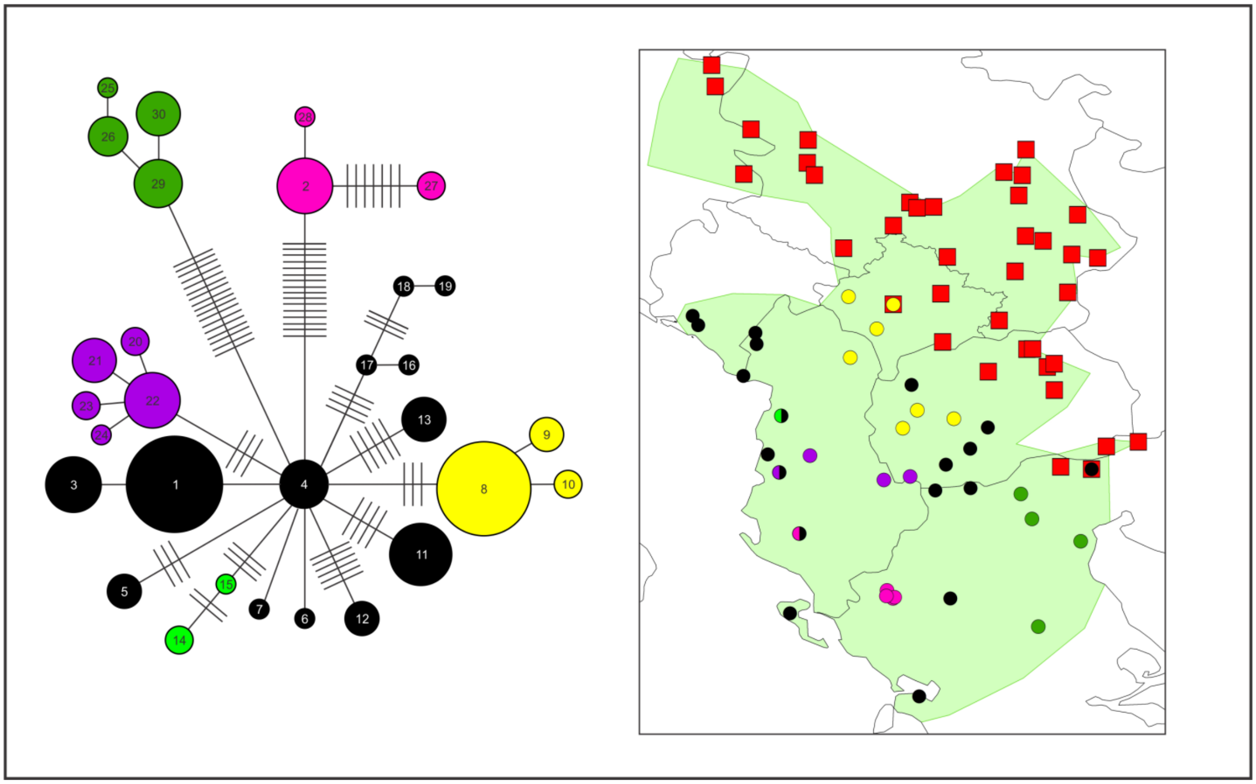

The haplotype network on the left represents *T. macedonicus* mitochondrial DNA. Pie diameter expresses haplotype frequency (cf. Additional file 5) and bars the number of substitutions along a branch if more than one. The numbers in the haplotype network refer to *T. macedonicus* mitochondrial DNA haplotypes as coded in Additional file 5. Color codes correspond to the *T. macedonicus* mitochondrial DNA phylogeny above and the map to the right. The map shows the spatial distribution of the groups of haplotypes that are significantly supported in the *T. macedonicus* mitochondrial DNA phylogeny above; haplotypes for which no interspecific structuring is revealed in the phylogeny are noted as black on the map (and in the haplotype network). The *T. macedonicus* populations that contain *T. karelinii* mitochondrial DNA are note on the map as red squares.
