# Supplementary material for: Postglacial species displacement in Triturus newts deduced from asymmetrically introgressed mitochondrial DNA and ecological niche models
Source: BMC Evol Biol. 2012 Aug 30;12:161. doi: 10.1186/1471-2148-12-161 (PMC3520116; doi:10.1186/1471-2148-12-161)
Supplement: Additional file 4 — Ecological niche modeling performance. The ecological niche models for Triturus macedonicus and T. karelinii tested against a null model. [file 1471-2148-12-161-S4.pdf]

Additional file 4: Ecological niche modeling performance. The ecological niche models for *Triturus macedonicus* and *T. karelinii* tested against a null model.

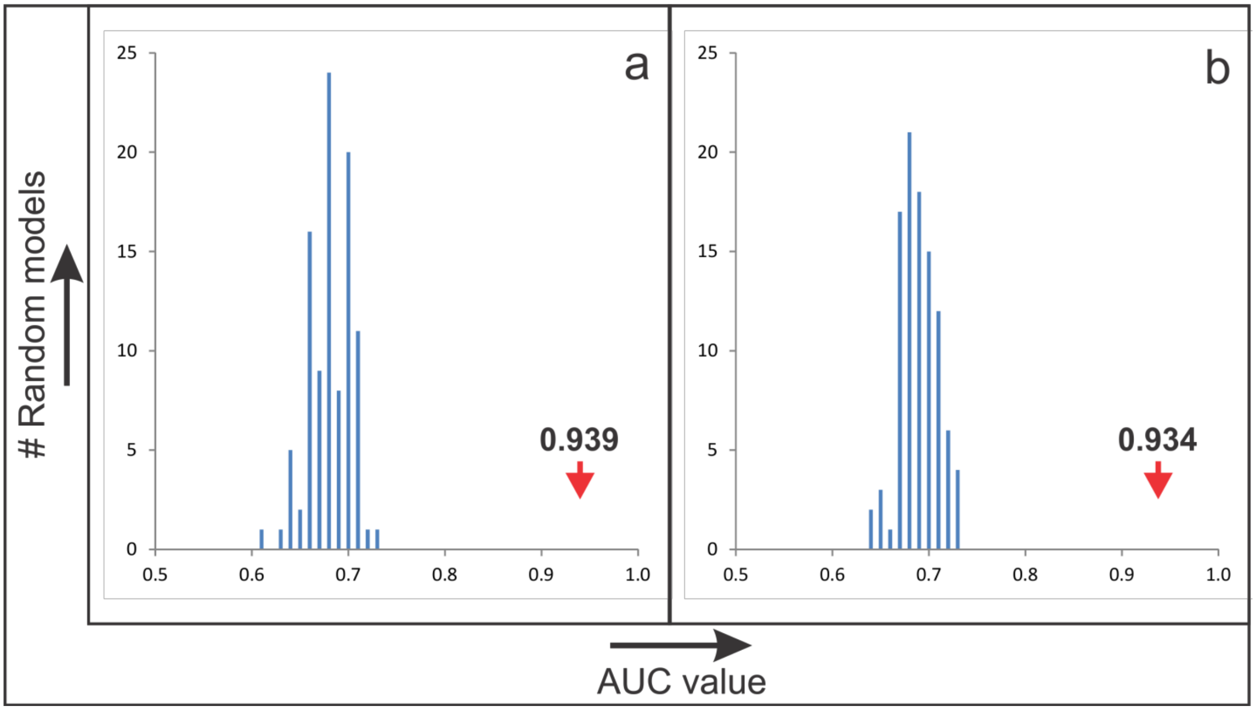

The ecological niche models preform statistically significantly better than random: the AUC values (marked with a red arrow) for *T. macedonicus* (a) and *T. karelinii* (b) fall to the right of a null distribution of AUC values, derived from 99 random models (based on as many random localities as are underlying the tested model).
