# Supplementary material for: Postglacial species displacement in Triturus newts deduced from asymmetrically introgressed mitochondrial DNA and ecological niche models
Source: BMC Evol Biol. 2012 Aug 30;12:161. doi: 10.1186/1471-2148-12-161 (PMC3520116; doi:10.1186/1471-2148-12-161)
Supplement: Additional file 5 — Contribution of bioclimatic variables to the ecological niche models. Response curves, contribution and permutation importance and results of a jackknife test for the bioclimatic variables. [file 1471-2148-12-161-S5.pdf]

Additional file 4: Contribution of bioclimatic variables to the ecological niche models. Response curves, contribution and permutation importance and results of a jackknife test for the bioclimatic variables.

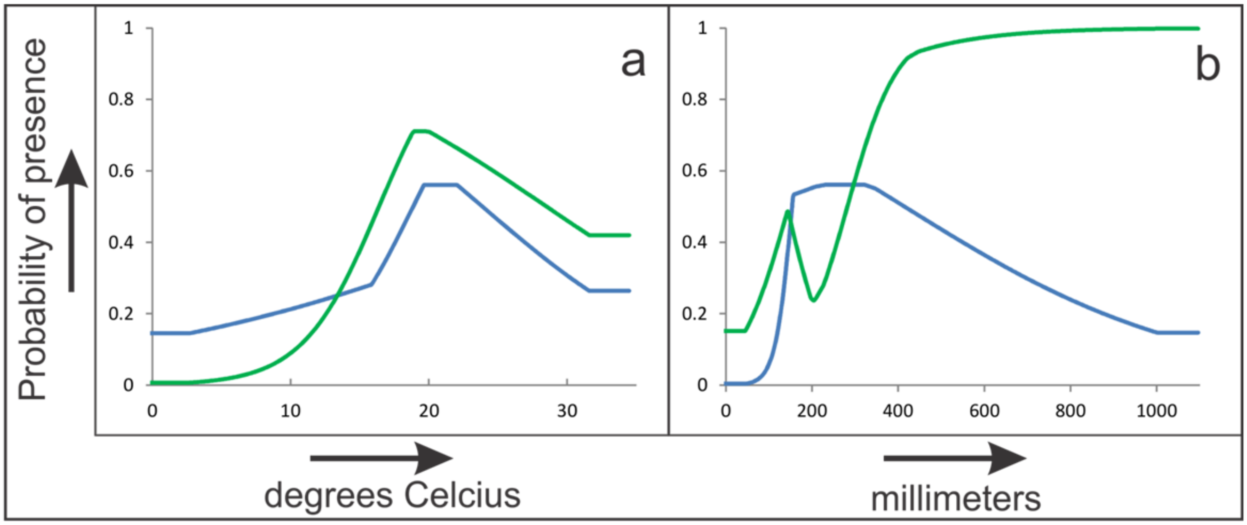

The response curves for the two bioclimatic variables that contribute most to the ecological niche models of *T. macedonicus* (green) and *T. karelinii* (blue). Bioclimatic variables are: (a) bio11 = mean temperature of coldest quarter and (b) bio16 = precipitation of wettest quarter.

	Bioclimatic variable				
	Bio 10 = mean temperature of warmest quarter	Bio 11 = mean temperature of coldest quarter	Bio 15 = precipitation seasonality	Bio 16 = precipitation of wettest quarter	Bio 17 = precipitation of driest quarter
Triturus karelinii					
Contribution	12.3%	64.9%	11.0%	4.8%	7.1%
Permutation importance	4.3%	68.3%	7.8%	3.4%	16.2%
Training gain if excluded	1.33	0.84	1.26	1.35	1.27
Training gain if used in isolation	0.55	0.83	0.25	0.05	0.10
Triturus macedonicus					
Contribution	11.3%	49.9%	2.6%	23.1%	13.2%
Permutation importance	6.1%	45.9%	5.5%	27.0%	15.6%
Training gain if excluded	1.47	1.35	1.52	1.32	1.33
Training gain if used in isolation	0.33	0.82	0.16	0.53	0.37

The contribution and permutation importance of the different bioclimatic variables to the ecological niche models of *T. macedonicus* and *T. karelinii* and the results of the jackknife tests as determined by Maxent.
