# Supplementary material for: Postglacial species displacement in Triturus newts deduced from asymmetrically introgressed mitochondrial DNA and ecological niche models
Source: BMC Evol Biol. 2012 Aug 30;12:161. doi: 10.1186/1471-2148-12-161 (PMC3520116; doi:10.1186/1471-2148-12-161)

Additional file 5: Differences between the current and mid-Holocene climate layers. For each of the bioclimatic values used in the ecological niche modeling, cells with a higher value under current climate compared to the mid-Holocene are shown in red and cells with a lower value in blue.

bio10 = mean temperature of warmest quarter:

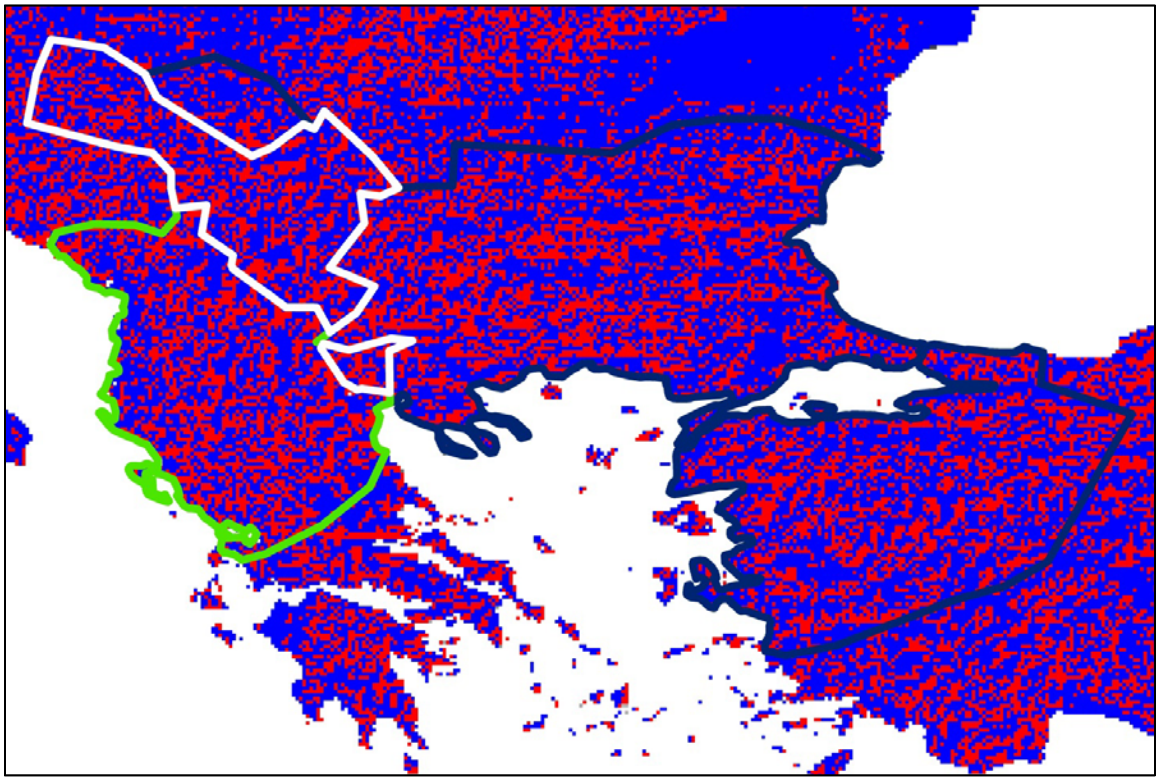

bio11 = mean temperature of coldest quarter:

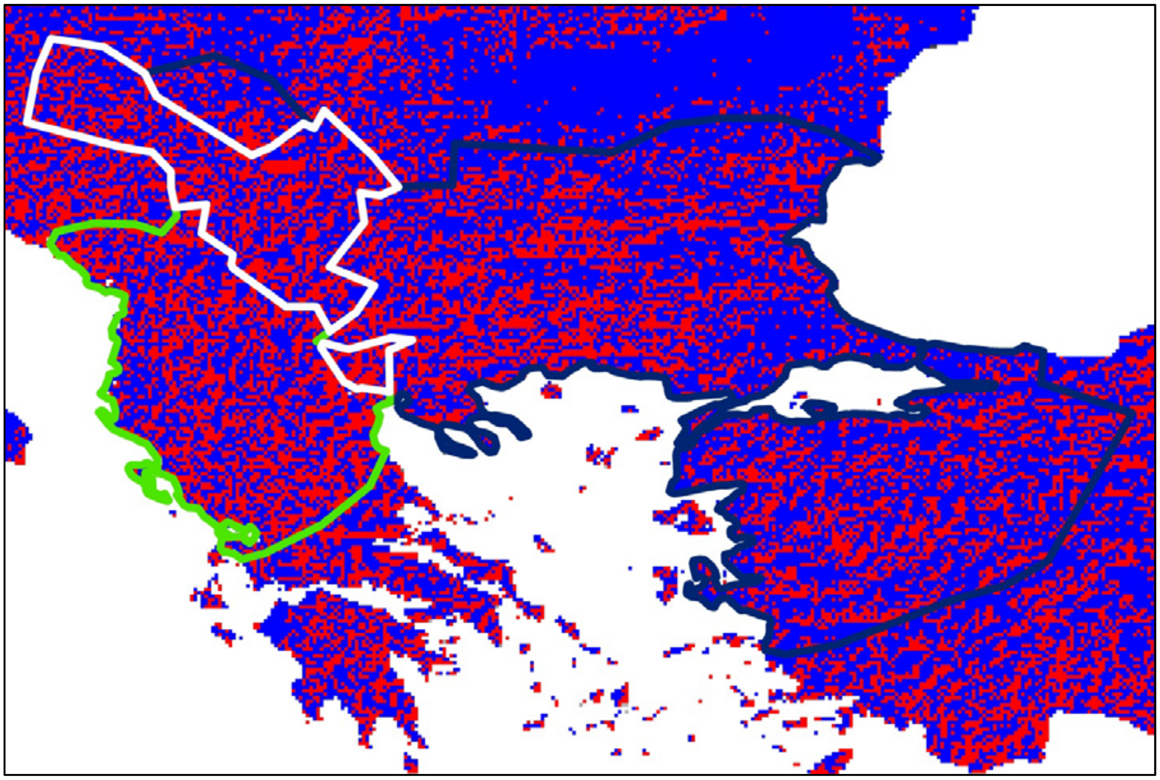

bio15 = precipitation seasonality:

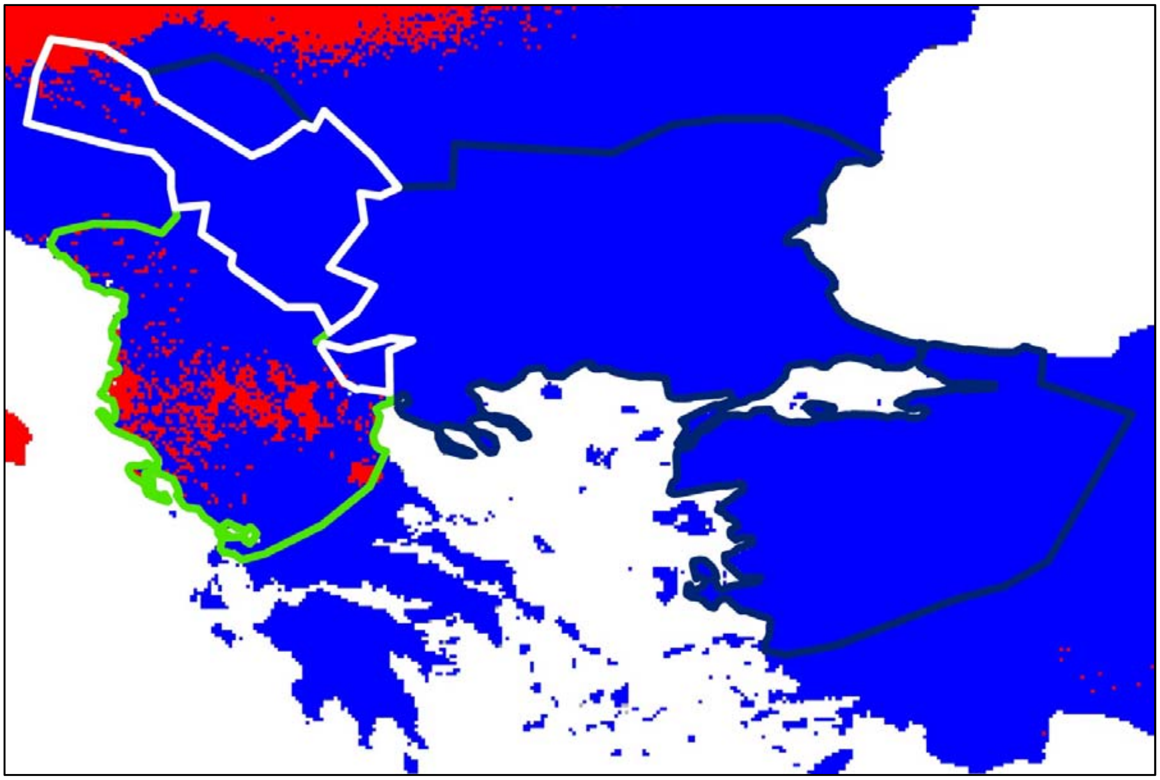

bio16 = precipitation of wettest quarter:

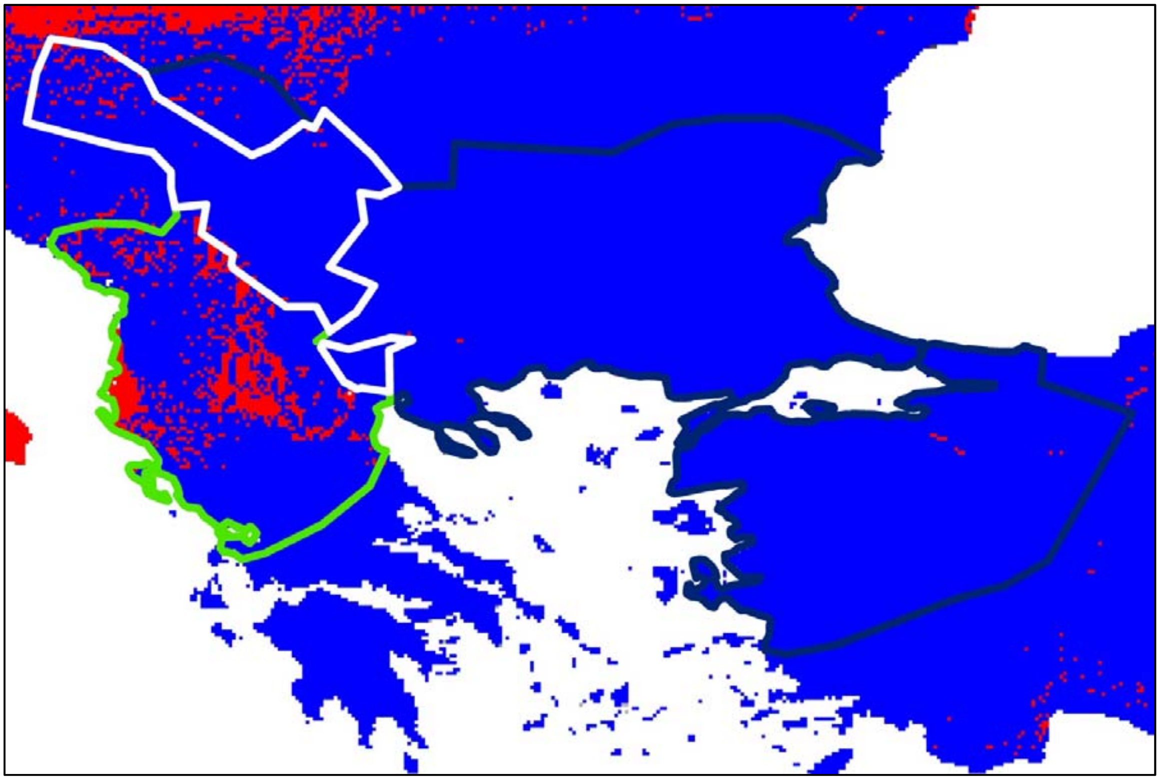

bio17 = precipitation of driest quarter:

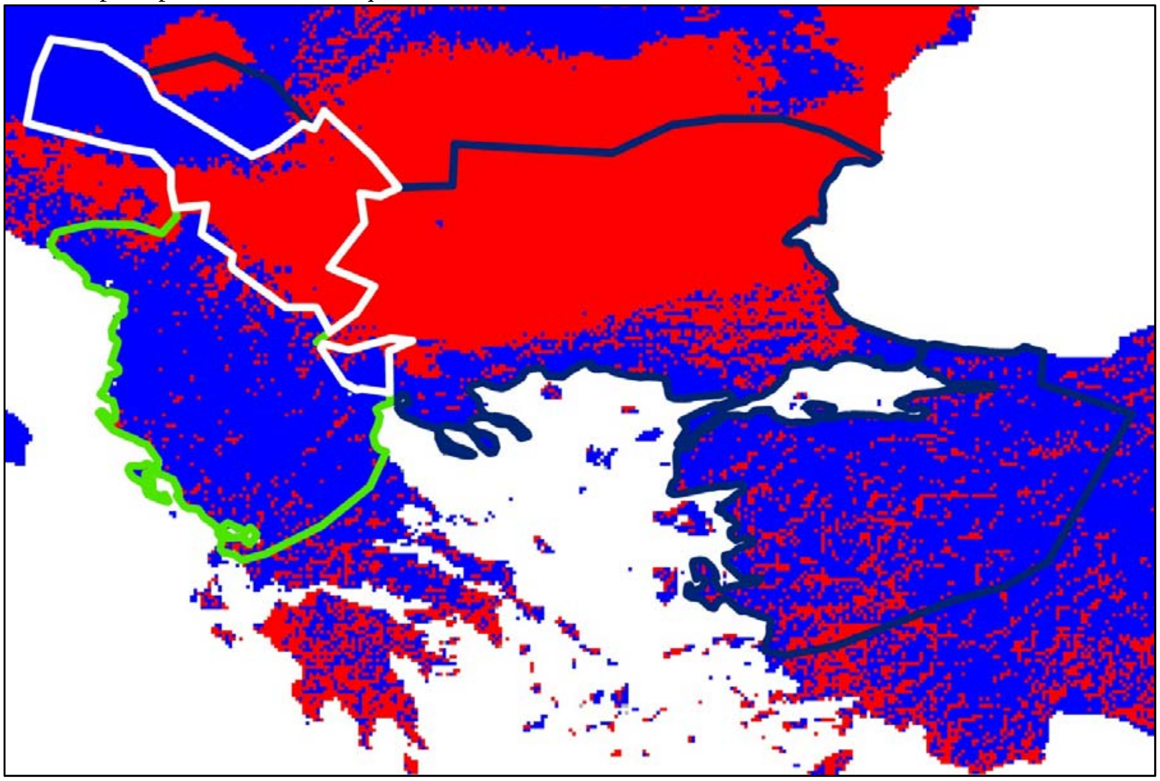

Supplement: Additional file 6 — Differences between the current and mid-Holocene climate layers. For each of the bioclimatic values used in the ecological niche modeling, cells with a higher value under current climate compared to the mid-Holocene are shown in red and cells with a lower value in blue. [file 1471-2148-12-161-S6.pdf]
